# Nuclear medicine imaging of posttraumatic osteomyelitis

**DOI:** 10.1007/s00068-016-0647-8

**Published:** 2016-02-17

**Authors:** G. A. M. Govaert, A. W. J. M. Glaudemans

**Affiliations:** 1 Department of Surgery, Subdivision of Trauma Surgery, University Medical Center Groningen, University of Groningen, Groningen, The Netherlands; 2 Department of Trauma Surgery, University Medical Center Utrecht, Utrecht, The Netherlands; 3 Department of Nuclear Medicine and Molecular Imaging, University Medical Center Groningen, University of Groningen, Hanzeplein 1, 9700 RB Groningen, The Netherlands

**Keywords:** Posttraumatic osteomyelitis, Nuclear medicine, FDG-PET, Bone scan, White blood cell scan

## Abstract

**Introduction:**

Early recognition of a possible infection and therefore a prompt and accurate diagnostic strategy is essential for a successful treatment of posttraumatic osteomyelitis (PTO). However, at this moment there is no single routine test available that can detect osteomyelitis beyond doubt and the performed diagnostic tests mostly depend on personal experience, available techniques and financial aspects. Nuclear medicine techniques focus on imaging pathophysiological changes which usually precede anatomical changes. Together with recent development in hybrid camera systems, leading to better spatial resolution and quantification possibilities, this provides new opportunities and possibilities for nuclear medicine modalities to play an important role in diagnosing PTO.

**Aim:**

In this overview paper the techniques and available literature results for PTO are discussed for the three most commonly used nuclear medicine techniques: the three phase bone scan (with SPECT-CT), white blood cell scintigraphy (also called leukocyte scan) with SPECT-CT and ^18^F-fluorodeoxyglucose (FDG)-PET/CT. Emphasis is on how these techniques are able to answer the diagnostic questions from the clinicians (trauma and orthopaedic surgeons) and which technique should be used to answer a specific question. Furthermore, three illustrative cases from clinical practice are described.

## Introduction

Osteomyelitis covers a wide range of bone infections caused by an infecting organism. Normally, bone is resistant to bacterial colonization; in trauma however the bone integrity can be disrupted by fractures, surgery or the presence of metal implants which makes it more vulnerable to exogenous microbial invasion. This, combined with the typically acute setting in which trauma surgery takes place with possibly contaminated open fractures and concomitant soft tissue injuries leads to a reported incidence of 1 to 19 % of deep infections after surgical fracture care. Not only this high infection rate is a concern but also due to an increase in surgical procedures over the last decades, fracture related osteomyelitis, also referred to as posttraumatic osteomyelitis (PTO), becomes more and more an entity that trauma—and orthopaedic surgeons will have to deal with [1, 2].

Essential for a successful treatment of PTO is an early recognition of the possible infection and therefore a prompt and accurate diagnostic strategy. A surgical site infection (SSI) occurs in the early phase (first 2 weeks after surgery) and can usually be recognized by clinical examination, since mostly the well-known four signs of an infection (swelling, redness, pain and heat) are present. In the later phases of PTO these signs may not be present and diagnosis can be difficult. It is however of invaluable importance to diagnose PTO as early as possible and to start early and specific treatment, since a late recognition or inadequate treatment may result in prolonged disease duration, high recurrence rate, high morbidity and sometimes even an amputation [3].

The diagnostic problem in PTO is that there is no single routine test available that can detect an infection with sufficiently high diagnostic accuracy. Mostly, a combination of clinical, laboratory, microbiological and medical imaging tests is performed [4] and the followed strategy depends on personal experience, tradition, financial aspects of the institute and best available evidence.

Diagnostic imaging routinely performed consists of plain X-rays and computed tomography (CT). These techniques are helpful to assess the position of metal implants and the union rate of the fracture, but are not able to differentiate between infection and inflammation. Magnetic resonance imaging (MRI) is better able to recognize infections; however, the metal implants can introduce artefacts and its diagnostic accuracy decreases after recent surgery as differentiation between sterile inflammation and infected tissue is difficult [5–7]. To our opinion, nuclear medicine imaging techniques play an important role in the diagnostic pathway to diagnose PTO. Nuclear medicine, which focusses at the pathophysiology of processes, is a booming area within the medicinal community. Pathophysiological changes usually precede anatomical changes, often leading to an earlier and possibly more accurate diagnosis. Recent developments in hybrid camera systems, combining the best of both anatomy and physiology with higher spatial resolution and better quantification possibilities, provides new opportunities and possibilities for these hybrid imaging modalities to play an important role in both diagnosis and therapy evaluation in patients with PTO.

The aim of this paper is to explain the existing nuclear medicine imaging possibilities for diagnosing PTO, how these modalities are able to answer the diagnostic questions from the clinicians (trauma and orthopaedic surgeons) and to provide an overview of which nuclear imaging technique should be used at which time point of the diagnostic pathway.

## Nuclear medicine in general; SPECT and PET

In nuclear medicine, radiopharmaceuticals (a radioactive element attached to a chemical compound or pharmaceutical specific for a disease process) are administered intravenously into the patient. As a result, images are performed from radiation which is emitted at the location of the disease/infectious process from within the patient. This characteristic forms the main distinction with radiology, which mainly focuses on tissue anatomy by using external radiation sources.

**Fig. 1:**
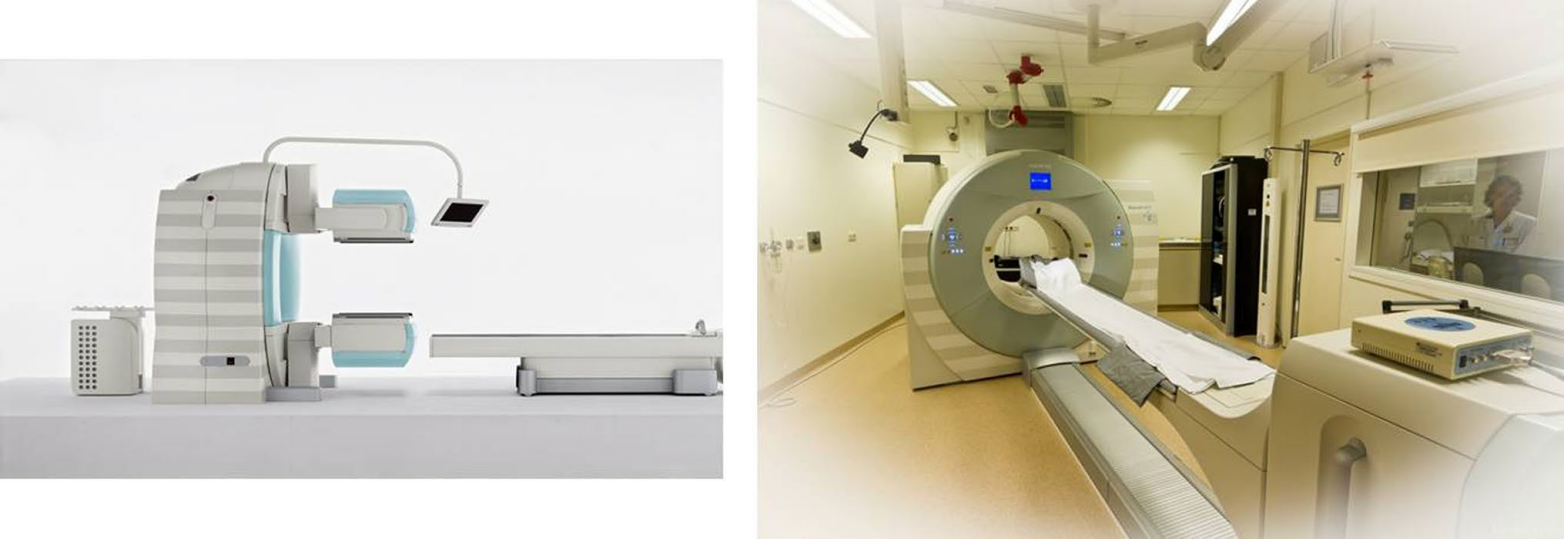
*Left image* gamma camera with SPECT-CT possibility (Siemens Symbia T).* Right* PET-CT camera (Siemens Biograph mCT 64-slice). Image courtesy: Siemens Medical Systems, Knoxville, TN

The two main camera systems used in nuclear medicine to visualize the radiopharmaceuticals are the gamma camera and the PET camera (Fig. 1). These camera systems detect the γ-rays emitted from the patient and transform it into an image (planar and/or 3D).

The already since the 1970s existing *gamma camera* forms the basis of conventional nuclear medicine by providing 2D planar imaging of the body. However, this technique has several limitations: image quality is rather poor and the spatial resolution of the gamma camera is limited to approximately 8 mm. Furthermore, it is difficult, based on 2D images with overlapping structures, to determine exactly where the increased uptake is located. The effect of this superposition can be overcome by collecting images from different angles (64 or 128) around the patient, thereby creating a 3D image. This technique, called *single photon emission computed tomography* (*SPECT*), leads to a higher contrast and improves sensitivity.

*Positron emission tomography* (*PET*) is a more recently (1990s) developed unique imaging tool to visualize various pathophysiological processes in the body. This technique is based on radionuclides that emit positrons (positively charged electrons) to become stable. Emitting positrons cannot exist freely, and therefore it meets his antimatter and annihilates into two γ-ray photons, each with the same energy and moving in opposite directions. The PET camera exist of a ring-shaped detector system which can detect the two photons when arriving within a certain time frame at opposite detectors. Recent developments in software lead to a correction method for the time a photon needs to travel from its origin to the detector. This software development, called time-of-flight (TOF) has major advantages for spatial resolution.

The major advantage of PET above SPECT is that that PET camera system has a greater efficacy in detecting photons, and a better spatial resolution of around 3–4 mm. Furthermore, quantification possibilities are better with PET.

## Added value of hybrid imaging; SPECT/CT and PET/CT

As already mentioned, recent development in both soft- and hardware led to the implementation of hybrid systems, combining SPECT and PET with CT. Both the nuclear medicine and radiological technique are performed in an immediate sequential setting, without changing the position of the patients, leading to an almost perfect correlation of pathophysiological with anatomical information. Furthermore, costs are reduced (one imaging modality), and the one-stop-shop principle (one combined scan instead of two separate scans at two different departments) reduces waiting time for the patient.

Very recently, PET systems were also combined with MRI, thereby introducing the PET/MRI hybrid imaging system. In these PET/MRI systems, the different modalities can be used in a simultaneous setting. PET/MRI has several major theoretical advantages that could be of interest for the whole medical community [8]. At the moment, this modality is mainly used in neurology and cardiology and its role in infectious processes in the musculoskeletal system has to be established.

## Nuclear medicine techniques to image PTO

Many radiopharmaceuticals are available to image infectious and inflammatory processes [9]. Only the worldwide most commonly used nuclear medicine techniques to image PTO will be discussed here by explaining the technical details of the procedure supplemented with a brief overview of the relevant literature. Finally, we will provide some illustrative clinical examples.

### Bone scintigraphy

#### Technique

Bone scintigraphy is one of the oldest existing nuclear medicine techniques and still one of the cornerstones in nuclear medicine practice. Radiopharmaceuticals used for bone scintigraphy are diphosphonates coupled to the radionuclide Technetium-99 m (^99m^Tc). These bone-seeking radiopharmaceuticals selectively accumulate on the surface on bone mineral matrix in areas of high metabolic activity and therefore depict osteoblastic activity.

**Fig. 2:**
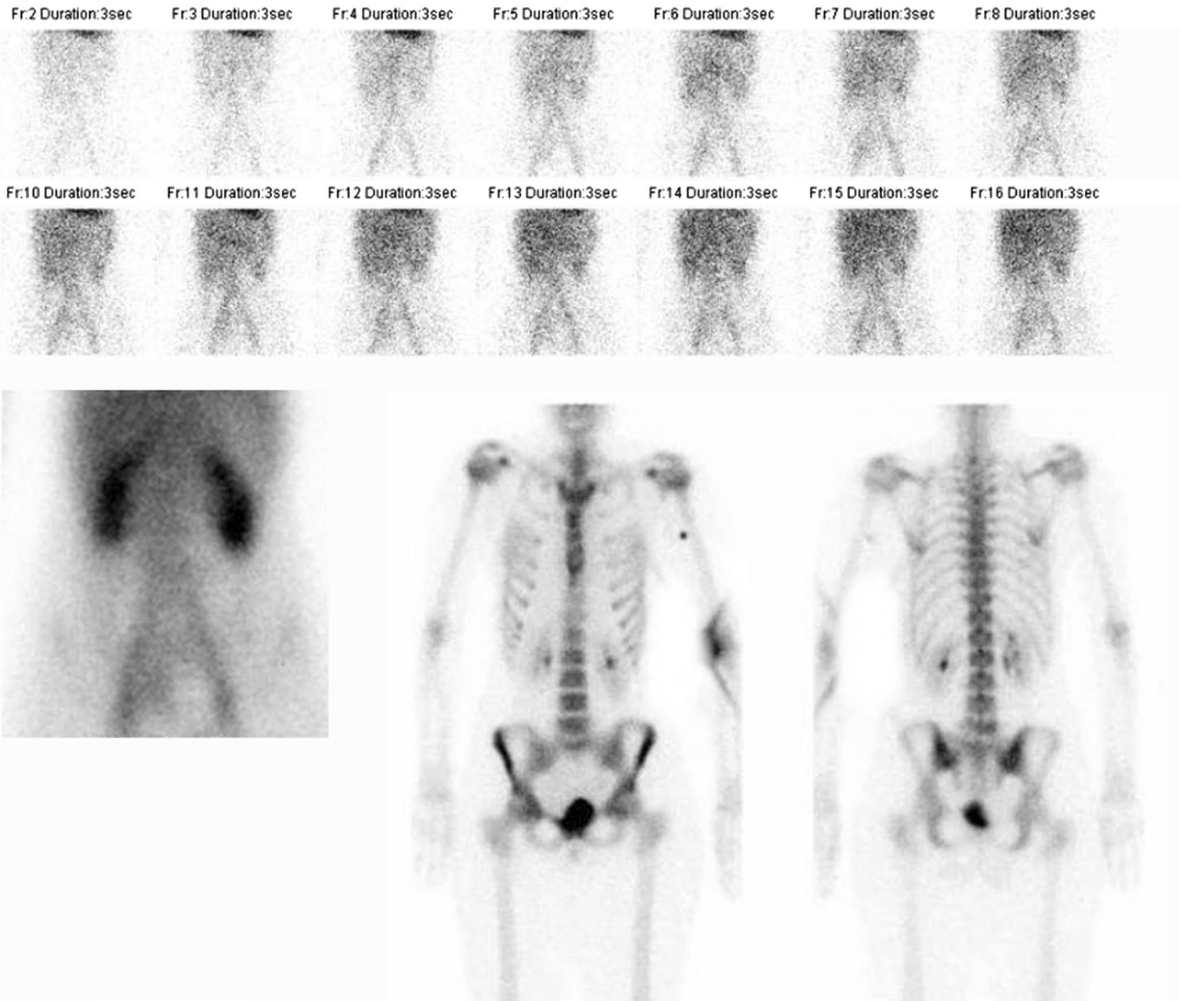
Example of a normal three-phase bone scan in a patient with pain complaints of the lumbar spine. *Upper row images*: flow/perfusion images (phase 1). *Lower row*, *left image* blood pool image (phase 2). *Lower row*, *middle image* (anterior view, phase 3), *right image* (posterior view, phase 3)

When a musculoskeletal infection is suspected, a three phase bone scintigraphy can be performed as a first screening tool (Fig. 2). As revealed by its name, this bone scintigraphy consists of three phases. The first phase is the perfusion phase, or flow study, performed dynamically, over the part of interest, for the first 2 min after administration of the radiopharmaceutical. The second phase is the blood pool phase, also performed on the part of interest, directly after the first phase (2–5 min after injection). The third phase, also called the static phase, depicts the incorporation of the radiopharmaceutical into the matrix of the bone and is usually performed 3 h after administration. This late phase can be combined with a SPECT-CT to localize the area(s) of increased bone metabolism. All three phases are necessary in case of suspected bone infection, since the three phases characterize both the vascularization and the metabolic activity of a process.

#### Bone scintigraphy in PTO

The three-phase bone scan can be used as a first screening method for diagnosing PTO. Because of its good availability it can mostly be performed short (<24 h) after the request of the referring clinician and it is relatively cheap. A normal bone scan (no increased perfusion and blood pool, no uptake in the late phase) rules out almost completely an existing bone infection (high sensitivity). The role of the bone scintigraphy, however, in the acute setting is neglectable, since the specificity is rather low and uptake is visible in all sites of increased bone metabolism irrespective of the underlying cause. A positive bone scan with an increased vascularity and increased metabolic uptake may indicate PTO; yet it can also indicate healing fracture(s) or a postsurgical situation. Furthermore, in a low-grade infection even the first two phases can be negative, so the late phase is essential and when positive it may be the only indication of an infection. Literature studies trying to find out at which time point a bone scan becomes negative after fractures and/or surgery are scarce. It is known that a bone scan may be positive for at least 2 years after total hip arthroplasty (THA) and 5 years after total knee arthroplasty (TKA) due to physiological bone remodeling after implantation [5]. We do not know exactly the time frame in which the bone scan is definitely positive following trauma, fracture or after open reduction and internal fixation (ORIF) of a fracture. Probably this time period will be around 1–2 years.

In conclusion, there is no role for a bone scintigraphy for diagnosing a SSI or early PTO. There is probably a role (when negative it excludes an infection) in the long-standing PTO, but a positive bone scintigraphy must be interpreted with caution and other imaging methods are necessary to differentiate between an infection and other causes of increased osteoblastic activity.

#### The “better” bone scan

The conventional bone scan as mentioned above is still the gold standard in bone imaging. The images are acquired on a gamma camera and most newer camera systems have also the possibility to include SPECT-CT in the imaging process. However, there is also a PET tracer for bone imaging which uses the radiopharmaceutical ^18^F-sodium fluoride (^18^F-NaF). The uptake mechanism of ^18^F-NaF resembles that of ^99m^Tc-labelled diphosphonates. The faster blood clearance and the twofold higher uptake in developing bone cells of fluoride make it possible to image faster (1 h after injection) and lead to better ratios between pathological and physiological bone uptake [10]. The advantages of using this PET tracer is the better resolution and better quantification possibilities. Limitations, however, are the higher costs and the lower availability worldwide of this techniques, and the non-possibility to perform flow and blood pool imaging. At this moment, the classical bone scan with labelled diphosphonates remains the gold standard when a bone scan is indicated; the ^18^F-NaF-PET could be considered for the individual patient.

### White blood cell (WBC) scintigraphy

#### Technique

Scintigraphy using labelled autologous white blood cells (WBC scintigraphy or leukocyte scintigraphy) was developed in the 1970s and is still the gold standard nuclear medicine technique for infections in the musculoskeletal system. It is a specific indicator for leukocyte infiltration into infected bone and soft tissue and is highly specific, since the WBCs accumulate by active migration to the infection. Over time, there have been major developments regarding how to correctly acquire, analyze and interpret the images, which eventually led to a high diagnostic accuracy. Also the possibility to better anatomically localize the infection due to the addition of SPECT-CT helped reach these good results mentioned in the literature.

**Fig. 3:**
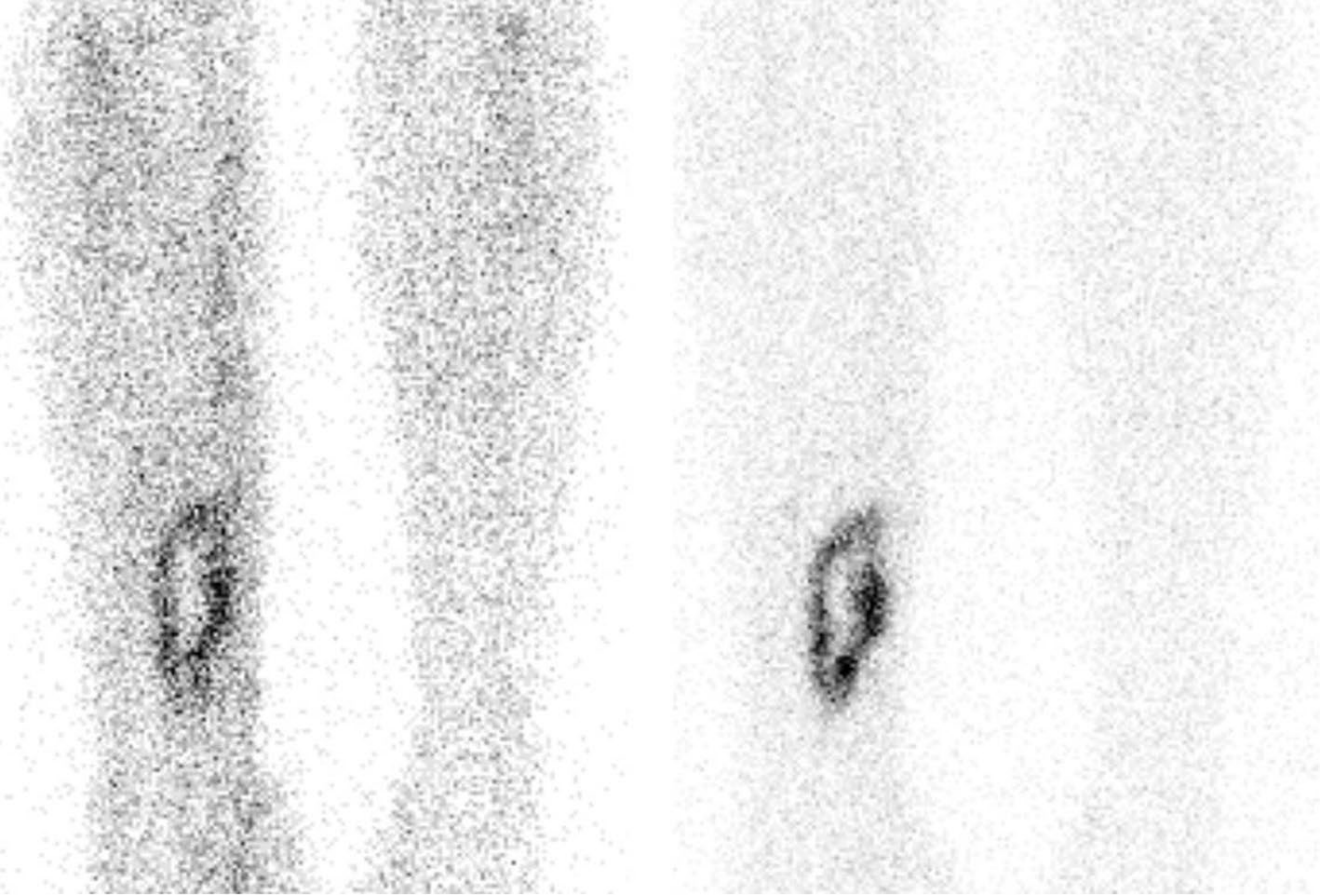
Example of a positive WBC scintigraphy of a 39 years old patient with osteomyelitis of the right tibia. *Left image* anterior view 4 h after injection. *Right image* anterior view 24 h after injection. Increase in uptake in time, especially when the background uptake is taking into account: suspect for an infection

Despite the high diagnostic accuracy, the whole procedure itself has limitations. First of all, 50–100 cc of blood has to be collected from the patient. Then, the preparation of the labelled (preferably with ^99m^Tc-HMPAO) white blood cells is laborious and time consuming (2–3 h) and must be performed under sterile conditions and strict regulations [11]. Subsequently, the labeled autologous leukocytes are reinjected into the patient. Finally, at least two imaging time points are necessary: 3–4 h after reinjection and 20–24 h after reinjection. This dual-time point imaging has to be performed since the accumulation of leukocytes in the infection is a dynamic process: it is the increase in size or intensity in time that indicates the presence of an infection (Fig. 3). When there is a decrease or the uptake is stable in time, then there is no infection but inflammation or physiological bone marrow uptake [8]. This change in uptake in time can be determined visually, but sometimes semi-quantitative evaluation can be a helpful tool as an addition to visual assessment. This is done by calculating ratios between the infectious focus and the contralateral side as background. Again, increase of the ratio in time points to an infection. Due to disintegration of the used radionuclide (^99m^Tc), the total acquisition time of the late images has to be prolonged accordingly to the half-life of the tracer to establish identical image quality. In accordance with the bone scintigraphy, a SPECT/CT can be performed to exactly localize the leukocyte uptake. The proposed correct procedure of acquisition and analysis of the scans are stated in a recent publication [12].

#### White blood cell scintigraphy in PTO

The role of WBC scintigraphy in peripheral osteomyelitis is extensively studied. Prandini et al. described in a meta-analysis of published papers up to December 2005, in almost 3600 cases, a diagnostic accuracy of 89 % [13]. In the included studies, however, different acquisition protocols and interpretation criteria were used. Furthermore, SPECT-CT did not exist at that time. So, probably the diagnostic accuracy is even higher when using the correct and standardized protocols and add the complementary information obtained by SPECT-CT. This was confirmed in two recent retrospective studies using these correct protocols in, respectively, 61 and 31 patients with peripheral osteomyelitis. The diagnostic accuracy in these studies was found to be very high: 97 and 100 %, respectively [12, 14].

The difficulty with interpreting the literature on the accuracy of WBC scintigraphy for diagnosing PTO is that most of these studies included patients with peripheral skeletal infections in general (including haematogenous osteomyelitis and prosthetic joint infections). Recently, we performed a systematic review of the recent literature (2000–2015) on the role of imaging modalities in patients with PTO (data not published yet). Only studies were included in which data for at least 10 patients with PTO were available, and a valid reference test (proven by histology or bacteriology, and/or clinical follow-up of more than 6 months) was described. Unfortunately, only 10 studies could be included of which 5 were performed with WBC scintigraphy (only one study reported a diagnostic accuracy, which was 98 %). So, despite the extensively available data of WBC scintigraphy in peripheral osteomyelitis in general, there is a lack of studies really focusing on PTO. Despite this disappointing finding, we believe that there is an absolute role for WBC scintigraphy in diagnosing PTO. This is based on expert opinion and best available evidence on osteomyelitis in general since it is the only existing imaging modality that is a specific indicator of an infection. Therefore, we retrospectively reviewed the diagnostic value of WBC scintigraphy ± SPECT/CT in 114 patients with suspected PTO in our hospital. Sensitivity, specificity, positive predictive value and negative predictive value were 89, 95, 86 and 97 %, respectively (data to be published).

### ^18^F-Fluorodeoxyglucose (FDG)-PET

#### Technique

**Fig. 4:**
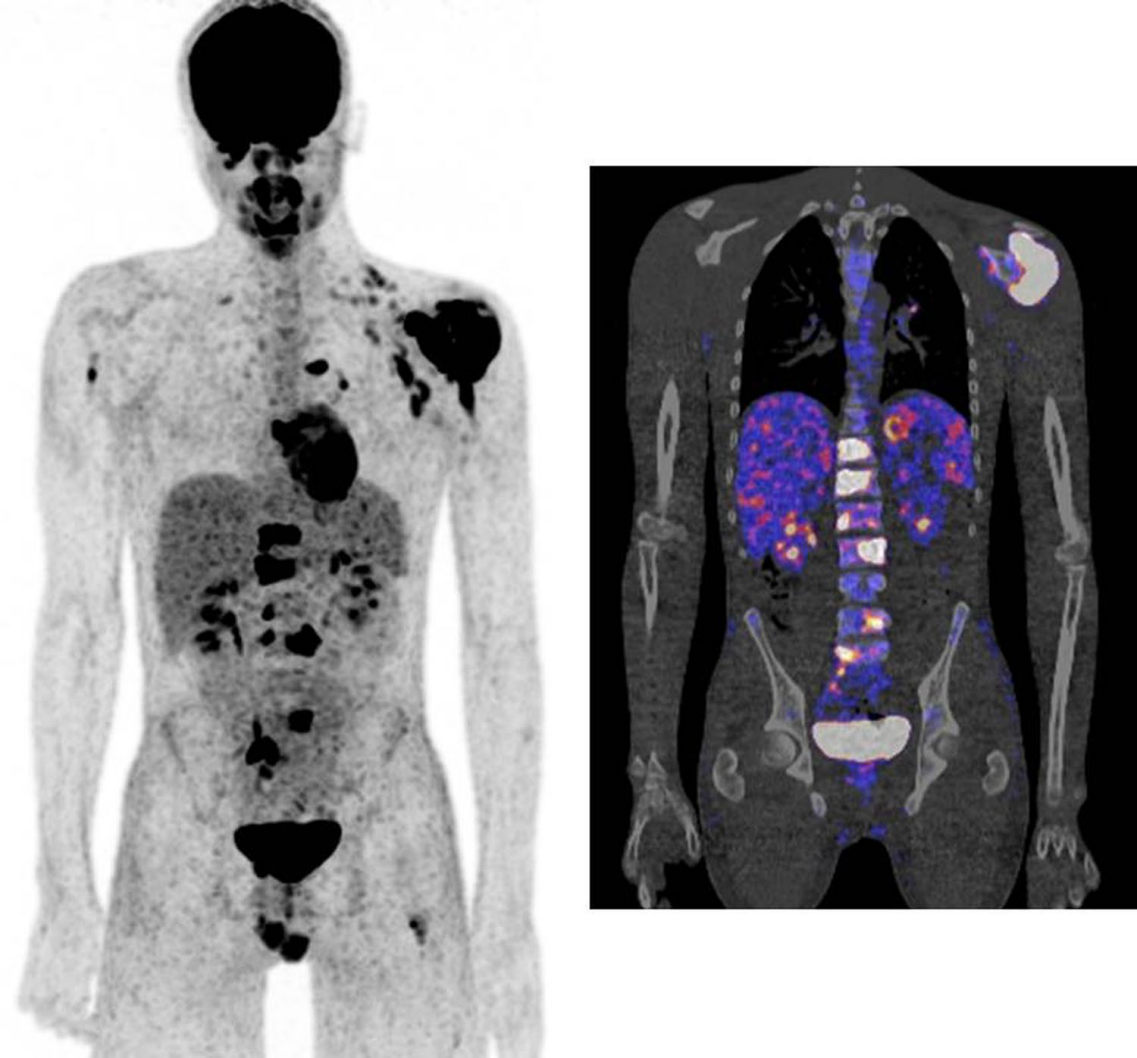
Patient with a proven tuberculosis osteomyelitis of the left shoulder. FDG-PET was performed to identify any disseminated foci of infection. FDG-PET image (*left*) and fusion PET-CT image (*right*) showing multiple infectious foci in the body (left shoulder, multiple vertebras, right upper arm, left upper leg)

The glucose analogue FDG is already extensively used in oncology for over a decade. It can also be used in infectious diseases, because activated leukocytes, monocytes, lymphocytes, macrophages and giant cells all use glucose as their energy source. To minimize FDG uptake in normal tissue, patients must fast for at least 4–6 h to reduce competition for glucose transporters on the cell membrane. After the injection of the labelled FDG (^18^F-FDG) patients must rest for an hour and limit physical activity to minimize muscle uptake and obtain a good biodistribution in the body. High contrast images of infectious lesions can be obtained with this technique. The use of FDG-PET has many advantages: no blood manipulation, high spatial resolution, one imaging time point which is already 60 min after injection, one-stop-shop possibility with diagnostic CT, etc. It is therefore an essential tool when searching for an infection or inflammation in a patient with fever of unknown origin or to establish the source of dissemination of infectious lesions in the body in a patient with an haematogeneous spread of the infection (Fig. 4).

Unfortunately, the uptake in both inflammatory and infectious cells makes this technique also very aspecific as it is often not possible to discriminate between inflammation and infection. No universal accepted interpretation criteria are available to declare a FDG-PET scan positive or negative for an infection, let alone for PTO. Furthermore, uptake of FDG in the peripheral skeleton results in a rather broad differential diagnosis. Osteomyelitis, soft tissue infection, inflammation, granulation tissue after surgery, reactive uptake around foreign body material, atherosclerosis, recent fractures and neuro-osteoarthropathy all lead to increased FDG uptake.

#### FDG-PET in PTO

As is the case with WBC scintigraphy, most data are available for FDG-PET imaging in long-standing (chronic) peripheral osteomyelitis. Termaat et al. performed a systematic review and meta-analysis for the assessment of chronic osteomyelitis and found the best results for FDG-PET with a sensitivity of 96 % and a specificity of 91 % [15]. Similar results were found by Jamar et al., who pooled all available data (in total 287 cases) and found a diagnostic accuracy of 94.5 % [16]. However, most of these studies dealt with chronic osteomyelitis from a diverse etiology and the exact role of FDG-PET in the posttraumatic situation is not known. Theoretically, due to reactive inflammation, the performance may be worse with osteosynthesis in situ, as is the case in prosthetic joint infections.

In the same systematic review our group performed as mentioned earlier, only 6 FDG-PET studies (with or without combined CT scan) were identified that were published in the last 15 years and fulfilled the criteria of >10 patients with suspected PTO and a valid reference test. The reported diagnostic accuracies were between 67 and 91 % (data not published yet).

In 2013, a shared guideline for the use of FDG in inflammation and infection was published by both the European Association of Nuclear Medicine (EANM) and the Society of Nuclear Medicine and Molecular Imaging (SNMMI) in the United States. This guideline states that the evidence for the use of FDG in osteomyelitis remains low (2b at best), and that at this moment, WBC scintigraphy is the preferred imaging modality. If FDG-PET is used however it is best performed in the chronic peripheral non postoperative setting [16].

## Questions from surgeon’s

In daily clinical practice in patients with suspected PTO, the trauma and orthopaedic surgeons have in general the following questions for the medical imaging specialists:

### Is the involved bone viable and are there sequestra?

To answer this question, a three-phase bone scintigraphy (preferably combined with a SPECT/CT) is required to see if there is perfusion and osteoblastic activity of the involved bone. In the case of suspected PTO, this is the only inquiry that is to be answered by bone scintigraphy. However, as said before: image quality is rather poor and the spatial resolution of the gamma camera is limited to approximately 8 mm, smaller sequestra can therefore be missed. When there is uptake, in the setting of PTO, it is often increased in intensity due to recent fracture or surgery, healing osteoblastic activity or osteomyelitis. Other imaging methods are then necessary to differentiate between these possibilities for the increased uptake.

### Is there an infection?

In patients with suspected PTO, with recent fracture and/or surgery, and osteosynthesis materials in situ, WBC scintigraphy is required. Increased uptake, increasing in size or intensity in time, indicate the presence of an infection.

In the late phase, and we do not know exactly the time point after fracture, probably 1–2 years, and without osteosynthesis in situ, FDG-PET is the best option, since this modality is easier to perform, has a high spatial resolution and only one imaging acquisition is necessary.

### When there is an infection, where is it located: in the bone or in the soft tissues?

This question is easy to answer with the now existing hybrid camera systems. When available, always perform SPECT-CT when there is uptake visible at the WBC scintigraphy and always perform PET-CT when the FDG-PET modality is used. With the anatomical correlation it is easy to localize exactly the area of increased accumulation: in (osteomyelitis) or outside (soft tissue infection) the bone.

## When should we use which nuclear technique?

In our opinion, these are the preferred nuclear imaging techniques for answering the different questions in a patient with suspected PTO (partly adapted from [17]:

**Table:** 

*Non-union, is there vital bone*: Three phase bone scan with SPECT-CT
*Non-union, is there an infection*: WBC scintigraphy with SPECT-CT
*Suspected peripheral PTO, no surgery or surgery >6 months ago and no osteosynthesis materials in situ*: FDG-PET/CT
*Suspected peripheral PTO, osteosynthesis materials in situ, placement <2 years ago*: WBC scintigraphy with SPECT-CT
*Suspected peripheral PTO, osteosynthesis in situ, placement >2 years ago*: Three phase bone scan, followed by WBC scintigraphy with SPECT-CT if the bone scan is positive
*Suspected PTO located in the axial skeleton*: FDG-PET/CT
*Suspected dissemination of infectious foci*: FDG-PET/CT

## Examples from clinical practice

### Patient A

#### Clinical story

Patient A, a 37-year-old healthy male, underwent open reduction and internal fixation (ORIF) of an open fracture of his right distal tibia and fibula 22 years ago. This was complicated by posttraumatic osteomyelitis and resulted in multiple re-operations with debridements of the bone, removal of most hardware and free flap coverage of a soft tissue defect. He was referred to our hospital with a persistent clinical infection around his right distal tibia and a near wound breakdown of the scar. Medical imaging was requested (a) to confirm the diagnosis of osteomyelitis and (b) to determine the anatomical location of the suspected osteomyelitis.

#### Imaging results

**Fig. 5:**
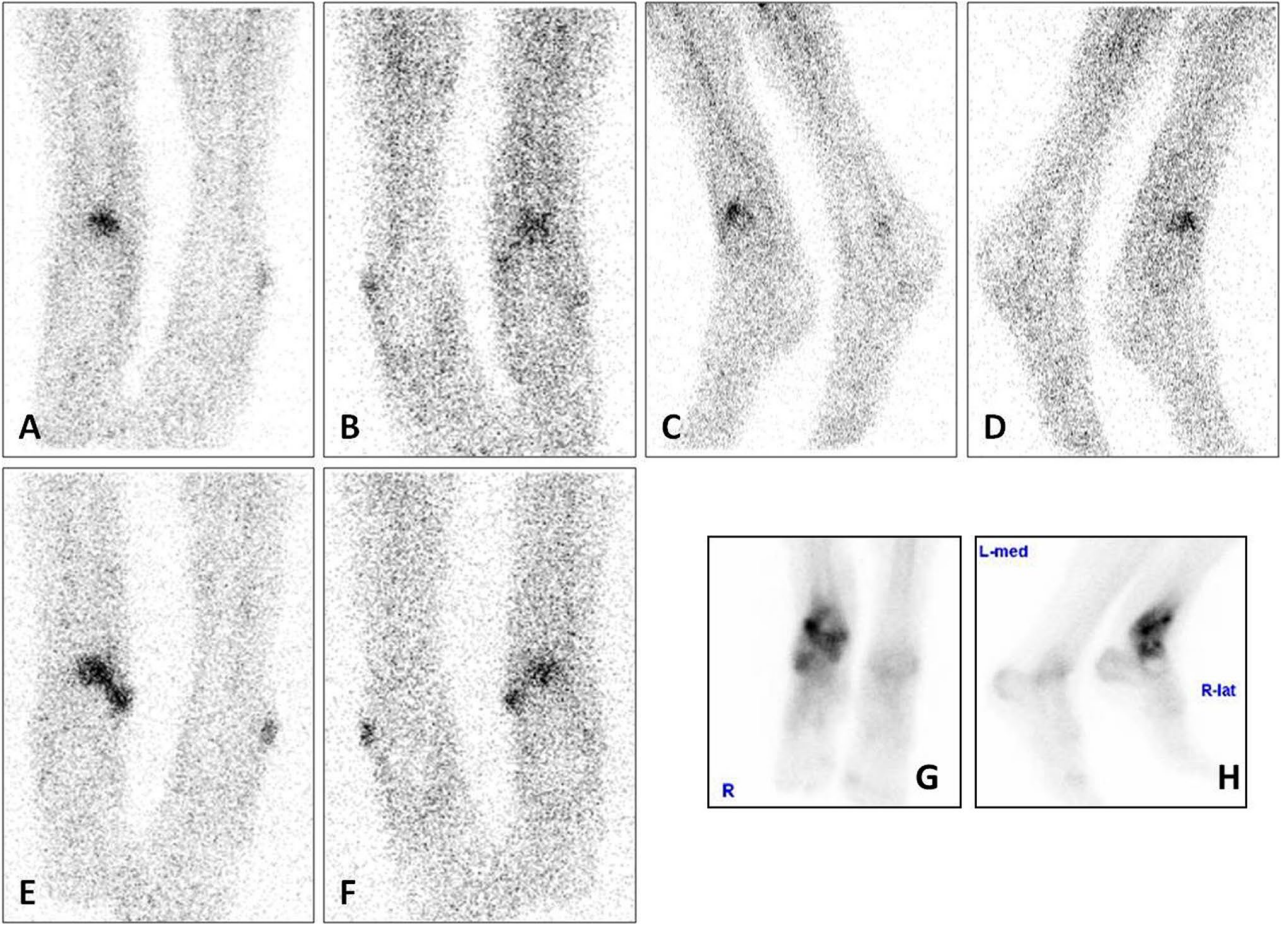
WBC scan (**a**–**d** images after 4 h, **e**–**f** images after 24 h) and late phase bone scan (**g** anterior view, **h** lateral view) of patient A

**Fig. 6:**
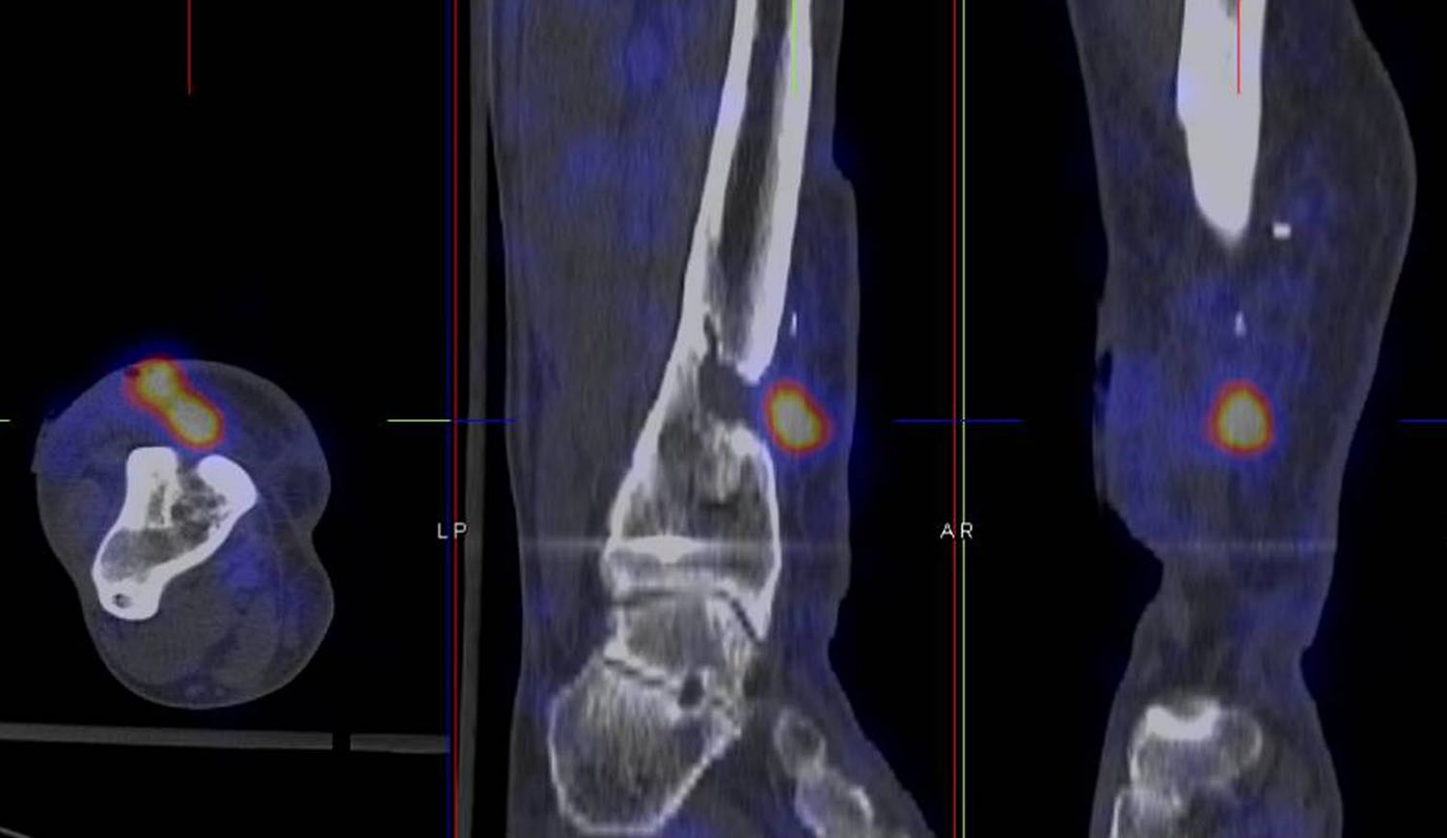
WBC scan SPECT-CT of patient A

First, according to the diagnostic imaging protocol in our hospital, a three-phase bone scan was performed since the fracture and surgery were >2 years ago. All three phases of the bone scan were positive, only the late phase (Fig. 5, g: anterior view, h: lateral view) is presented here. This increased osteoblastic uptake can be the result of an infection, but also due to a healing fracture or recent surgery. For further differentiation, the patient underwent a WBC scan (Fig. 5, a–d: images after 4 h, e–f: images after 24 h). This showed increased uptake in both intensity and size over time, suspect for an infection. To localize this accumulation of leukocytes a SPECT-CT was performed (Fig. 6) which revealed that the uptake was located outside the bone, in the soft tissue. Final diagnosis was a soft tissue infection.

### Patient B

#### Clinical story

**Fig. 7:**
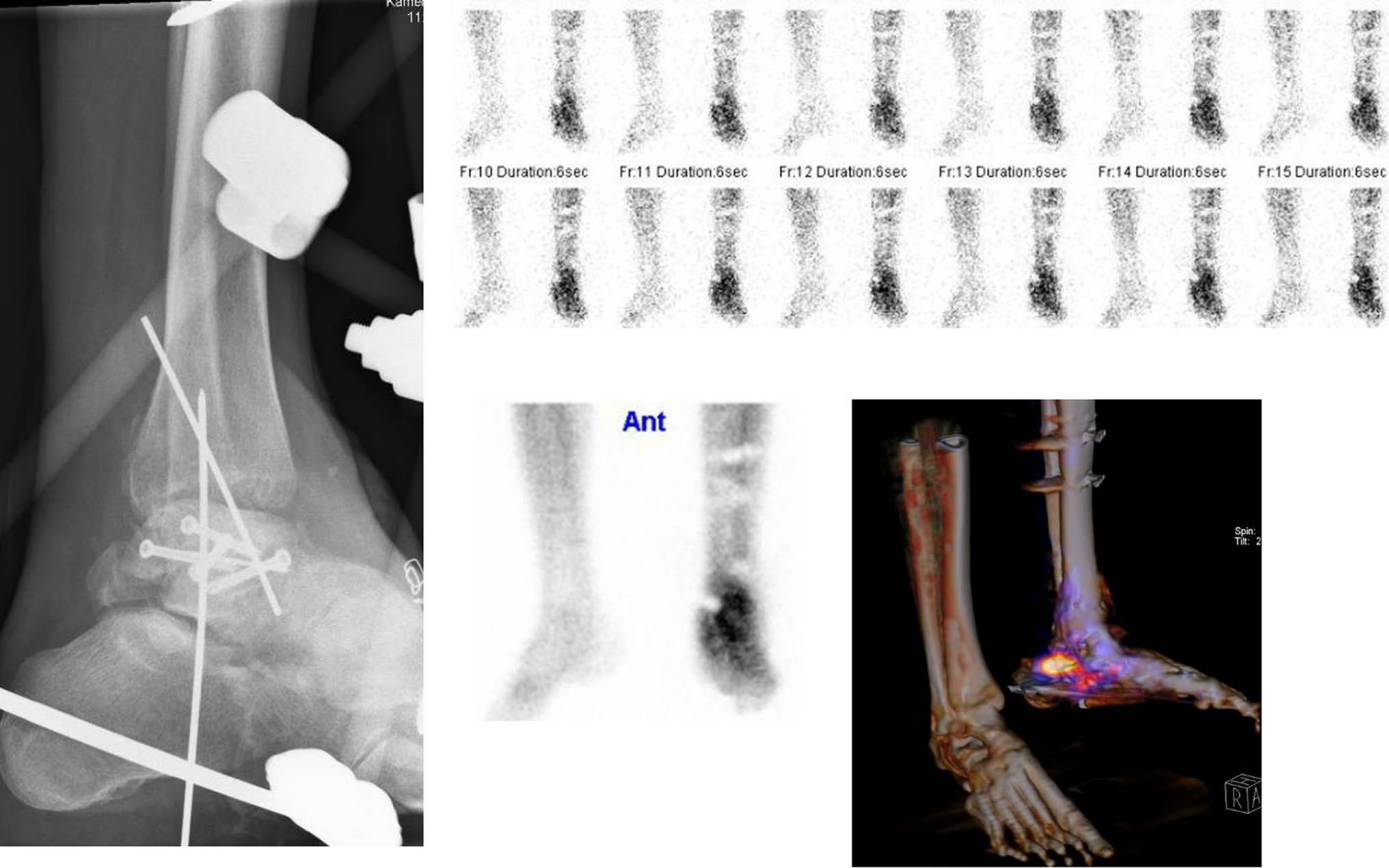
X-ray (*left image*) and bone scan (*upper row* flow phase, *middle image lower row* blood pool phase, *right image lower row* SPECT-CT late phase) of patient B

Patient B, a 46-year-old schizophrenic but otherwise healthy male sustained an open and comminuted talar neck fracture after a fall from height. This was initially treated with multiple soft tissue debridements and an external fixation, later augmented with screw and K-wire fixation of the fracture. The soft tissue defect was closed with a local myocutaneous flap. Two months after this last procedure, the patient presented with a draining sinus in the scar on the lateral side of the ankle joint. Medical imaging was requested to (a) assess the viability of the talus and (b) to determine the anatomic location of the suspected osteomyelitis. The X-ray is shown in Fig. 7 (left image).

#### Imaging results

**Fig. 8:**
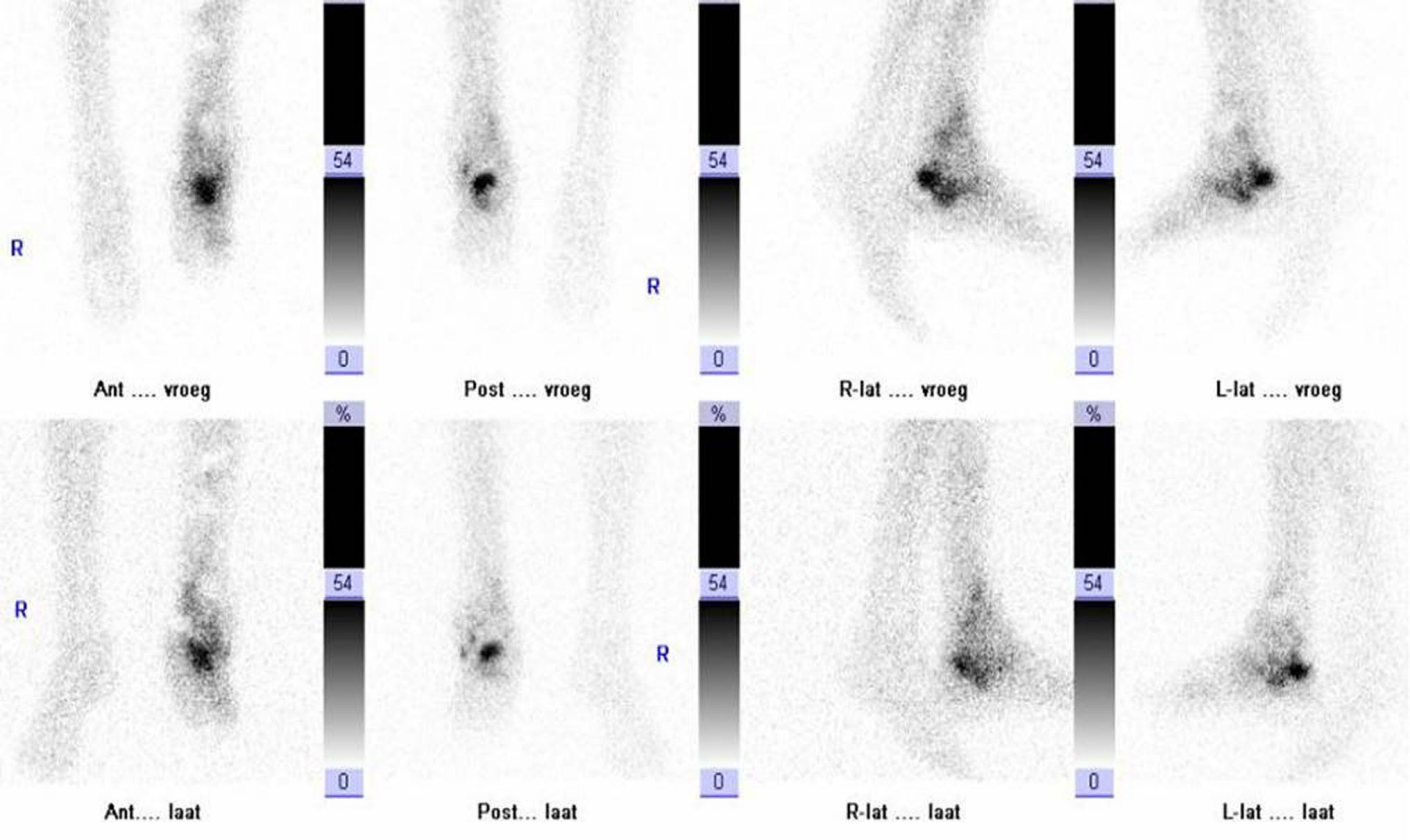
WBC scan (*upper row* images after 4 h, *lower row* images after 24 h) of patient B

To answer question (a) a three phase bone scan was performed. The first phase (flow phase) is shown in Fig. 7 (upper row images): positive flow at the talar region of the left foot. Obviously also the second phase (blood pool phase, middle image lower row) and the late phase (combined with CT image, right image lower row) are positive. This means the bone is viable. However, differentiation between infection or healing fracture is not possible. Therefore, WBC scintigraphy was performed (Fig. 8, upper row: images after 4 h, lower row: images after 24 h). The uptake decreased in time, meaning that the leukocyte accumulation is the result of a healing fracture and not of an osteomyelitis. After proper wound care further healing was uneventful.

### Patient C

#### Clinical story

Patient C, a 33-year-old healthy male was referred to our hospital because of a suspected posttraumatic osteomyelitis in combination with a malunion of his left tibia. He sustained a gunshot wound to his left lower leg in the middle east conflict 2 years prior to this presentation which was treated with a prolonged immobilization in an external fixator combined with several wound debridements. The last operation was only a few months prior to presentation. On examination, apart from the obvious malalignment of his left lower leg, we noted a closed but unstable scar on the medial side of his left tibia. Medical imaging was requested to (a) confirm the diagnosis and (b) to determine the anatomic location of the suspected osteomyelitis.

#### Imaging results

**Fig. 9:**
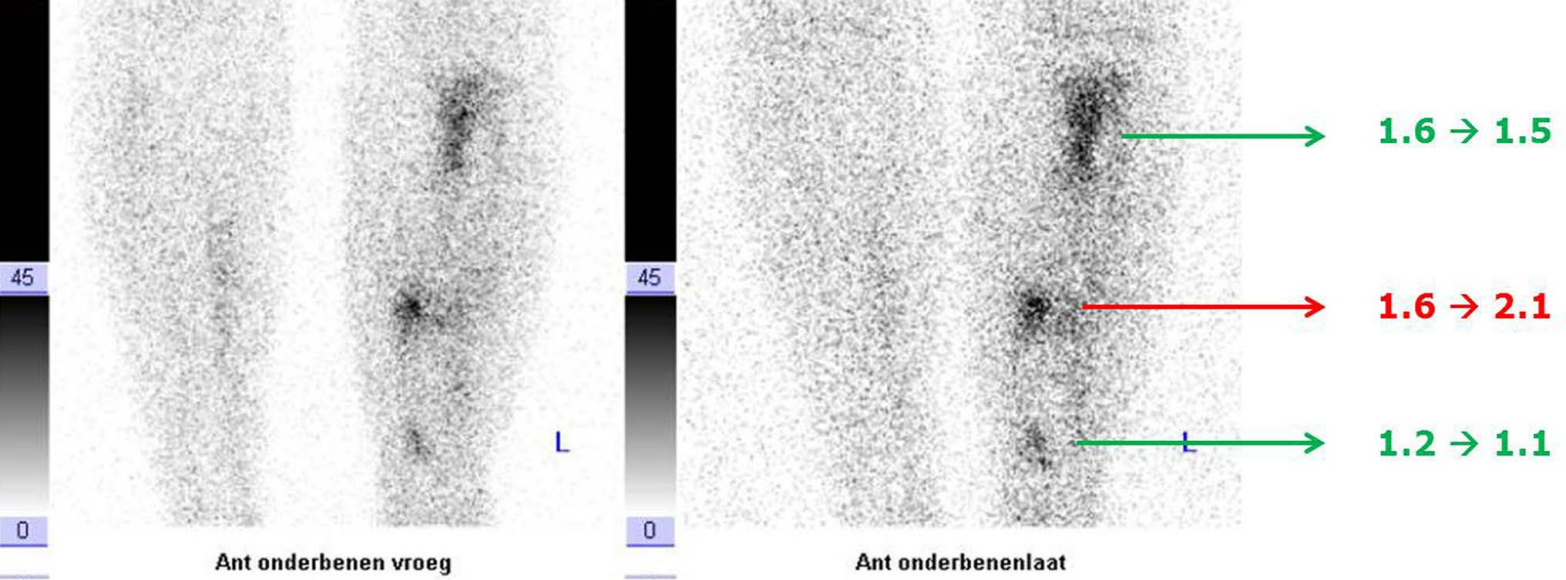
WBC scan (*left image* anterior view after 4 h, right image: anterior view after 24 h) of patient C

**Fig. 10:**
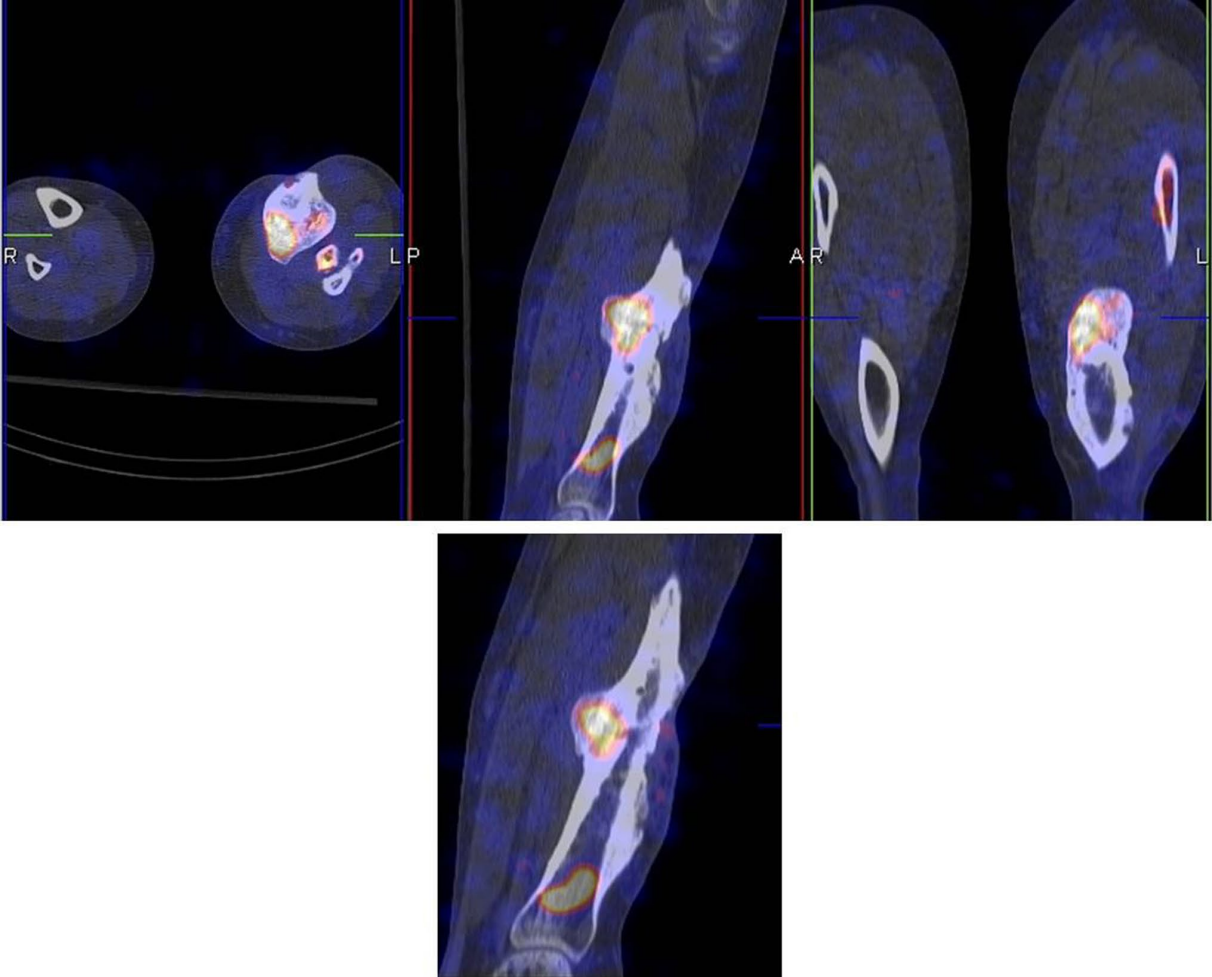
WBC SPECT-CT of patient C

Since his last surgery was <6 months ago, immediately a WBC scan was performed (Fig. 9, left image: anterior view after 4 h, right image: anterior view after 24 h): uptake is visible at three locations. When calculating the ratios (uptake focus-to-contralateral side) the uptake at the most proximal and most distal focus decreases in time. This means these uptakes are due to regeneration of bone marrow. However, the uptake at the middle focus increases in time, which is suspect for an infection. The SPECT-CT (Fig. 10) shows the uptake in the bone and a small fistula to the bone marrow. Indeed, surgery revealed an infection at this location.

## Conclusion

Nuclear medicine modalities play an important role in the assessment of posttraumatic osteomyelitis. Three phase bone scintigraphy can be used to exclude PTO in longstanding cases, but when positive other imaging techniques are necessary. WBC scintigraphy, when using the correct acquisition, analysis and interpretation protocols, is a specific technique to diagnose an infection with high diagnostic accuracy. FDG-PET has several advantages and can perfectly be used in a chronic non postoperative setting, but should be interpreted with caution when metal implants are in situ or when surgery was performed recently. The pros and cons of the three different techniques are summarized in Table 1.

**Table 1:** Nuclear medicine modalities to image PTO

Imaging test	Major advantages	Major disadvantages	Relative costs (€)	Sensitivity/specificity	When to order	Other comments
Bone scan + SPECT/CT	Widely available Cheap Negative bone scan excludes infection	Low specificity: increased uptake at all sites of increased bone metabolism irrespective of the underlying disease No role in acute PTO Probably positive for 1–2 years after ORIF	300-400	Sensitivity 80–90 % Specificity 50–70 %	Viable bone Suspected peripheral PTO, osteosynthesis in situ, placement >2 years ago	Positive bone scan must be interpreted with caution and other imaging methods are necessary to differentiate between an infection and other causes of increased osteoblastic activity
WBC scan + SPECT/CT	Specific for leukocytic infiltration Accurately detects both acute and chronic infections High diagnostic accuracy	Laborious preparation Dual time point imaging necessary	800–1000	Sensitivity 80–100 % Specificity 80–100 %	Suspected infected non-union Suspected peripheral PTO, osteosynthesis materials in situ, placement <2 years ago; or when bone scan is positive >2 years	Correct acquisition, analysis and interpretation protocol has to be followed With SPECT-CT differentiation between osteomyelitis and soft tissue infection possible
FDG-PET/CT	Short acquisition time High image resolution No need for blood manipulation	Not possible to differentiate between infection and inflammation No existing criteria for positivity	1000–1200	Sensitivity 40–100 %* Specificity 60–90 %* *depending on which criteria for positivity are used	Suspected peripheral PTO, no surgery or surgery >6 months ago and no osteosyn-thesis in situ Suspected PTO in the axial skeleton Suspicion for dissemination	Consensus criteria for positivity necessary

Right: PET-CT camera (Siemens Biograph mCT 64-slice)

Image courtesy: Siemens Medical Systems, Knoxville, TN, USA

Prospective studies comparing these nuclear medicine imaging techniques with radiological imaging techniques like MRI are necessary to provide evidence based diagnostic flowcharts in patients with suspected PTO.
